# Simultaneous extraction and determination of alkaloids and organic acids in *Uncariae Ramulas Cum Unicis* by vortex-assisted matrix solid phase dispersion extraction coupled with UHPLC-MS/MS

**DOI:** 10.3389/fchem.2023.1100150

**Published:** 2023-01-25

**Authors:** Xianjun Xu, Jiake Wen, Shuangqi Wang, Jia Hao, Kunze Du, Shiming Fang, Jun He, Jin Li, Yanxu Chang

**Affiliations:** ^1^ Wuyishan Institute of biology, Nanping, Fujian, China; ^2^ State Key Laboratory of Component-based Chinese Medicine, Tianjin University of Traditional Chinese Medicine, Tianjin, China; ^3^ Haihe Laboratory of Modern Chinese Medicine, Tianjin, China

**Keywords:** Uncariae Ramulas Cum Unicis, response surface methodology, silica, UHPLC-MS/MS, vortex-assisted matrix solid phase dispersion

## Abstract

A simple and efficient vortex-assisted matrix solid phase dispersion with a ultra-high-performance liquid chromatography-triple quadrupole mass spectrometer (VA-MSPD-UHPLC-MS/MS) was applied for simultaneous extraction and determination of seven alkaloids and three organic acids from *Uncariae Ramulas Cum Unicis*. The optimal extraction conditions of the target components were obtained by Box-Behnken design (BBD) combined with response surface methodology (RSM). The results of the method validation showed that this analytical method displayed good linearity with a correlation coefficient (r) no lower than 0.9990. The recoveries of ten active ingredients from *Uncariae Ramulas Cum Unicis* ranged from 95.9% to 103% (RSD ≤ 2.77%). The RSDs of intra-day and inter-day precisions were all below 2.97%. The present method exhibited not only lower solvent and sample usage, but also shorter sample processing and analysis time. Consequently, the developed VA-MSPD-UHPLC-MS/MS method could be successfully and effectively used for the extraction and analysis of ten active components from *Uncariae Ramulas Cum Unicis*.

## Introduction

Traditional Chinese Medicines (TCMs) exert their curative effects through characteristics such as multi-component, multi-channel, and multi-target. However, it is difficult to clarify the pharmacodynamic substance present in TCMs due to their complex chemical components. At present, various studies have been devoted to seeking efficient extraction and analysis methods of the chemical constituents of TCMs, thereby laying a foundation for further promoting research on the material basis of the medicinal effects of TCMs (Wang et al., 2021). Gouteng (*Uncariae Ramulas Cum Unicis*), the hook-bearing branches of *Uncaria rhynchophylla* (Miq.) Miq. ex Havil, *Uncaria macrophylla* Wall., *Uncaria hirsuta* Havil., *Uncaria sinensis* (Oliv.) Havil., and *Uncaria sessilifrudus* Roxb, belongs to the Rubiaceae family according to the Chinese Pharmacopoeia 2020 (Chinese Pharmacopoeia Commission, 2020). In clinical treatment, it is usually used for the treatment of cardiovascular and nervous system diseases (Kushida et al., 2021). Various chemical components such as alkaloids, triterpenes, flavonoids, sterols, and phenols, etc., have been isolated from Gouteng (Xie et al., 2013). Current studies have revealed that Gouteng possesses anti-hypertensive (Huang et al., 2021), anti-convulsant (Shao et al., 2016), sedative and hypnotic (Sakakibara et al., 1998), anti-inflammatory, and anti-cancer (Chen et al., 2017) properties. These pharmacological activities are mainly related to the chemical constituents, especially in alkaloids which contains, for example, rhynchophylline, isorhychophylline, isocorynoxeine, corynoxeine, geissoschizine methyl ether, and so on (Qin et al., 2021). Therefore, it is of great significance for the quality control and clinical application of Gouteng to extract and determinate these active components using eco-friendly and efficient methods.

To date, methods for the separation and detection of active components from Gouteng mainly employ ultra-high-performance liquid chromatography coupled with Quadrupole-Orbitrap-mass spectrometry (UHPLC/Q-Orbitrap-MS) (Huang et al., 2021), gas chromatography-mass spectrometry (GC-MS) (Tan et al., 2011), and high-performance liquid chromatography coupled with a photodiode array (HPLC-PAD) (Kaiser et al., 2016). Ultrasonic-assisted extraction (UAE) is a commonly used method for extracting a number of components from Gouteng (Pan et al., 2015). However, this method not only requires a large amount of sample and organic solvent, but also takes a long time for sample processing. Matrix solid phase dispersion (MSPD) was first introduced by Barker in 1989 as a novel sample preparation method, with many advantages such as reducing sample and reagent consumption and lower costs (Barker, 2000). Recently, an increasing number of modified MSPD extraction methods have been established by researchers, including ultrasound-assisted MSPD (UA-MSPD) (Dos Santos et al., 2019), vortex-assisted MSPD (VA-MSPD) (de Melo Malinowski et al., 2022), and micro salting-out assisted MSPD (µ-SOA-MSPD) (Zhang et al., 2021), which simplify the extraction procedures of traditional MSPD and reduce both the loss of target components and the sample pretreatment time. Currently, these modified MSPD methods have been used for the extraction of active components from various herbs medicines such as terpenoids, flavonoids, and alkaloids (Wang et al., 2019; Chen et al., 2021; Zheng et al., 2021). The modified MSPD method is a good way to avoid the mutual transformation of isomeric alkaloids in Gouteng under heating conditions.

The selection of dispersant or adsorbent is a crucial parameter that influences the MSPD procedure (Capriotti et al., 2015). The appropriate dispersant/adsorbent can disrupt the sample structure, disperse analytes on the solid carrier, increase the efficiency of the interaction between the sample and solvent, and improve the extraction efficiency of the target components. Various materials have been applied as dispersants/adsorbents in the MSPD extraction procedure, such as C_18_, silica, alumina, florisil PR, and *β*-CD (Xu et al., 2016; Wang et al., 2017; Wang et al., 2018), as well as other novel materials such as a molecular sieve, molecularly imprinted polymers, and metal-organic frameworks (Cao et al., 2016; Yin et al., 2019; Zhang et al., 2020). Silica is cheap, easy to obtain, rich in hydroxyl groups, and possesses a porous structure. It has been used for the extraction of iridoid glycosides, anthraquinone, and catechins from TCM (Du et al., 2018). Therefore, silica can be used as an effective dispersant for the extraction of effective components from Gouteng.

In this work, an efficient and fast vortex-assisted matrix solid phase dispersion (silica-VA-MSPD) coupled with UHPLC-MS/MS method was established for simultaneous extraction and determination of seven alkaloids (isocorynoxeine, corynoxeine, isorhychophylline, rhynchophylline, geissoschizine methyl ether, hirsuteine, and hirsutine) and three organic acids (chlorogenic acid, neochlorogenic acid, and cryptochlorogenic acid) from Gouteng. In order to obtain an optimal extraction efficiency for the active components, the key parameters for the VA-MSPD-UHPLC-MS/MS method, including the type of dispersant, ratio of sample to dispersant, grinding time, concentration of extraction solvent, volume of extraction solvent, and vortex time were investigated by applying a single factor optimization experiment and a Box-Behnken design combined with response surface methodology (BBD-RSM).

## Materials and methods

### Chemicals and reagents

The reference standards isocorynoxeine, corynoxeine, isorhychophylline, rhynchophylline, geissoschizine methyl ether, hirsuteine, hirsutine, chlorogenic acid, nuciferine (IS), and rosmarinic acid (IS) were purchase from Chengdu DeSiTe Biological Technology Co., Ltd. (Chengdu, China). Neochlorogenic acid and cryptochlorogenic acid were provided by Chengdu Must Biological Technology Co., Ltd. (Chengdu, China) (Supplementary Figure S1). The purity of all reference standards was over 98%. MS-grade formic acid was purchased from Anaqua Chemicals Supply (United States). Methanol and acetonitrile, of HPLC grade, were supplied by Fisher Scientific (Pittsburg, PA, Uinted States) and Honeywell (China) Co., Ltd, respectively. Deionized water was obtained from a Milli-Q ultrapure water system (Millipore, United States).

### Plant materials

Fifteen batches of Gouteng were obtained from different provinces, and were authenticated as *Uncaria. spp* by Prof. Yanxu Chang (Tianjin University of Traditional Chinese Medicine). All samples were crushed into a powder and passed through a 50-mesh sieve. All samples were deposited at Tianjin State Key Laboratory of Modern Chinese Medicine (Tianjin, China).

### UHPLC-MS/MS conditions

**FIGURE 1:**
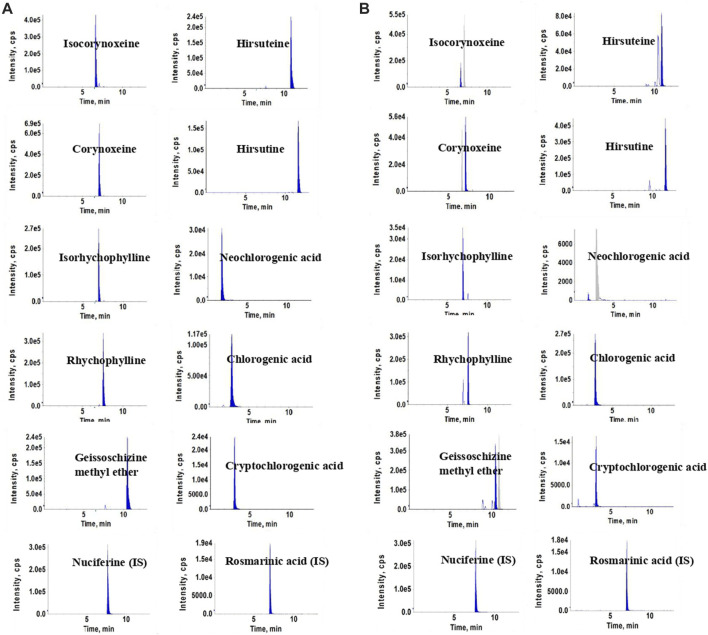
Chromatograms of the ten compounds and internal standards in standards solutions **(A)** and sample solutions **(B)**.

Experiments were performed on an Agilent 1290 UHPLC system (Agilent Corporation, United States) and an API 3200 triple-quadrupole mass spectrometer (Concord, Ontario, Canada). A ZORBAX Eclipse XDB-C18 (2.1 mm × 100 mm, 1.8-Micron, Agilent) was used for chromatographic separation. The mobile phase consisted of formic acid aqueous solution (0.1%, v/v) (A) and acetonitrile (B), with application of the gradient elution as follows: 0–5 min, 10%–26% (B); 5–7 min, 26%–27% (B); 7–11 min, 27%–35% (B); and 11–13 min, 35%–95% (B). The column temperature and injection volume were set at 35°C and 2 μL, respectively. The flow rate was maintained at 0.3 mL/min. The key parameters of the electric spray ion source (ESI) in positive and negative ion modes were optimized, and the optimal results were as follows: curtain gas (CUR), 15 psi; ion spray voltage (IS), ± 4500 V; ion source temperature (TEM), 550°C; gas1 (GS1), 45 psi; gas2 (GS2), 25 psi. The MS parameters of each compound, including the declustering potential (DP), entrance potential (EP), collision energy (CE), and collision cell exit potential (CXP) are shown in the Supplementary Table S1. Chromatograms of the ten compounds and internal standards, in sample solution and working standard solutions, respectively, are shown in Figure 1.

### Preparation of standard and internal standard (IS) solutions

All reference standards with a final concentration of 1.0 mg/mL, including isocorynoxeine, corynoxeine, isorhychophylline, rhynchophylline, geissoschizine methyl ether, hirsuteine, hirsutine, chlorogenic acid, neochlorogenic acid, and cryptochlorogenic acid, were precisely weighed and dissolved in methanol. All solutions were stored at 4°C before analysis.

### Silica-VA-MSPD procedure

**FIGURE 2:**
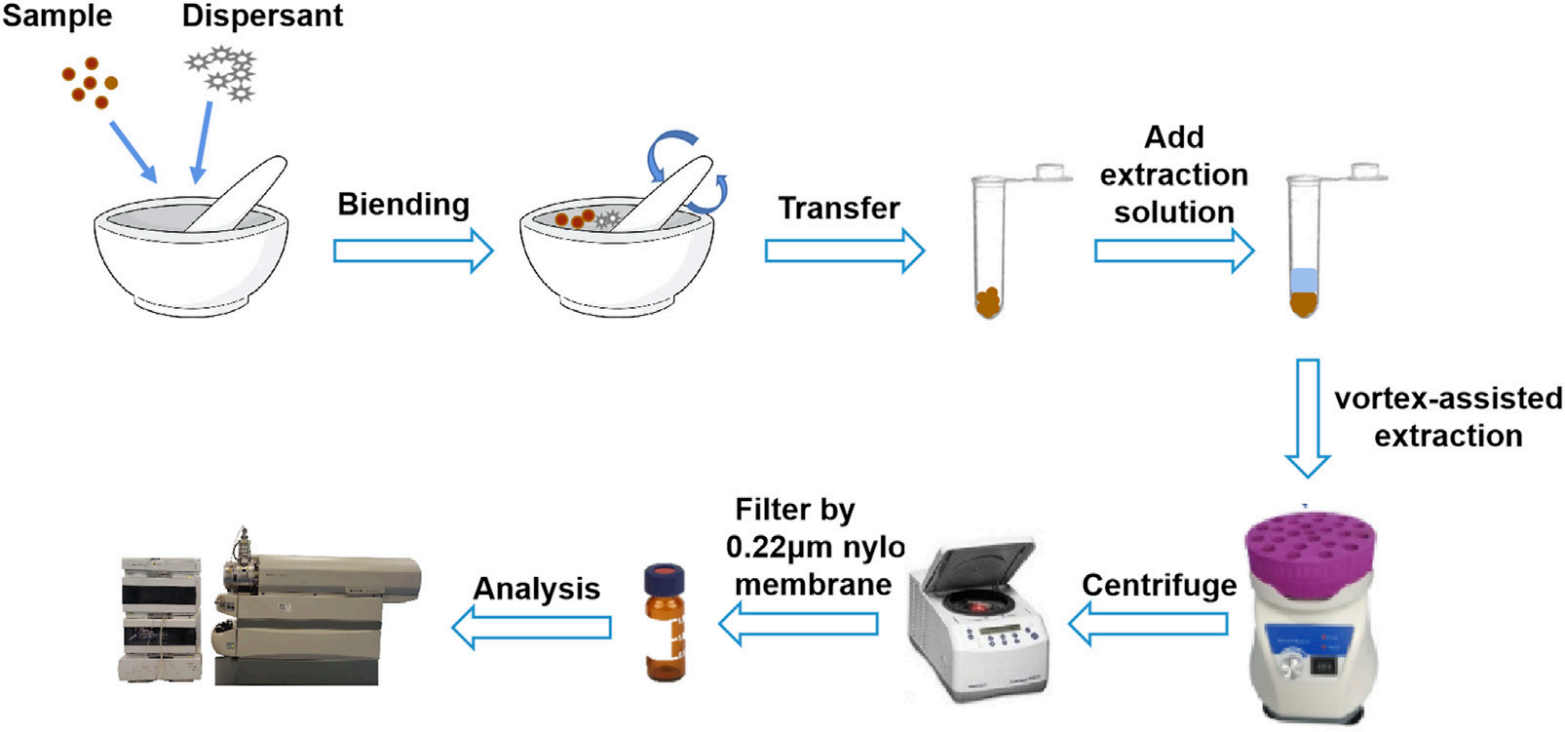
Schematic diagram of the VA-MSPD-UHPLC-MS/MS method.

Gouteng powder (20 mg) and dispersant (20 mg) were accurately weighed, placed in an agate mortar, and ground for 1 min until the mixture became homogeneous. Then, the mixture was transferred to a centrifugal tube and 1.3 mL 75% methanol was added. The resultant solution was extracted on a vortex mixer for 4 min and centrifuged at 14,000 rpm for 10 min. The supernatant was filtered through a 0.22 µm microporous filter membrane to obtain the sample solution. The sample solution was stored in a refrigerator at 4°C for subsequent analysis. A schematic diagram of the silica-VA-MSPD procedure is shown in Figure 2.

### Ultrasonic extraction

Gouteng powder (200 mg) was placed in a 50 mL Erlenmeyer flask and extracted with 8 mL methanol (70%, v/v) by ultrasonication for 45 min. The extracted solution was centrifuged at 14,000 rpm for 10 min and filtered through a 0.22 µm organic microporous filter membrane before analysis (Xie et al., 2013).

### Reflux extraction

0.50 g Gouteng powder was precisely weighed and transferred to a 100 mL round-bottomed flask and 60 mL ultrapure water was added. This solution was refluxed for 60 min. Then, the resultant solution was centrifuged and passed through a 0.22 µm filter membrane before injection analysis (Tan et al., 2011).

### Optimization of VA-MSPD parameters

VA-MSPD parameters, including type of dispersant, ratio of sample to dispersant, grinding time, concentration of extraction solvent, volume of extraction solvent, and vortex time, were individually investigated to acquire the optimum extraction yield of all active ingredients in Gouteng. Each parameter of VA-MSPD was tested as follow: dispersants including PCX, silica, SCX, and C_18_, ratio of sample to dispersant of 1:0, 1:1, 1:2, 1:3, and 1:4, grinding time of 0, 1, 2, and 3 min, extraction solvent of 25%, 50%, 75%, and 100% methanol, volume of extraction solvent of 0.5, 0.75, 1.0, 1.25, and 1.5 mL, vortex time of 1, 2, 3, 4, and 5 min. Each experiment was performed in triplicate.

### BBD coupled with RSM optimization experiment

To obtain the optimal extraction yields of the target components from Gouteng, BBD coupled with RSM was selected to optimize the three crucial parameters of VA-MSPD: extraction solvent (A), extraction solvent volume (B), and vortex time (C). Using Design Expert (version 8.0.6) software, BBD-RSM optimization of the three factors and the three levels was designed, and the corresponding experimental verification was carried out using the predicted optimal extraction conditions.

## Results and discussion

### Optimization of VA-MSPD parameters

#### Type of dispersant

**FIGURE 3:**
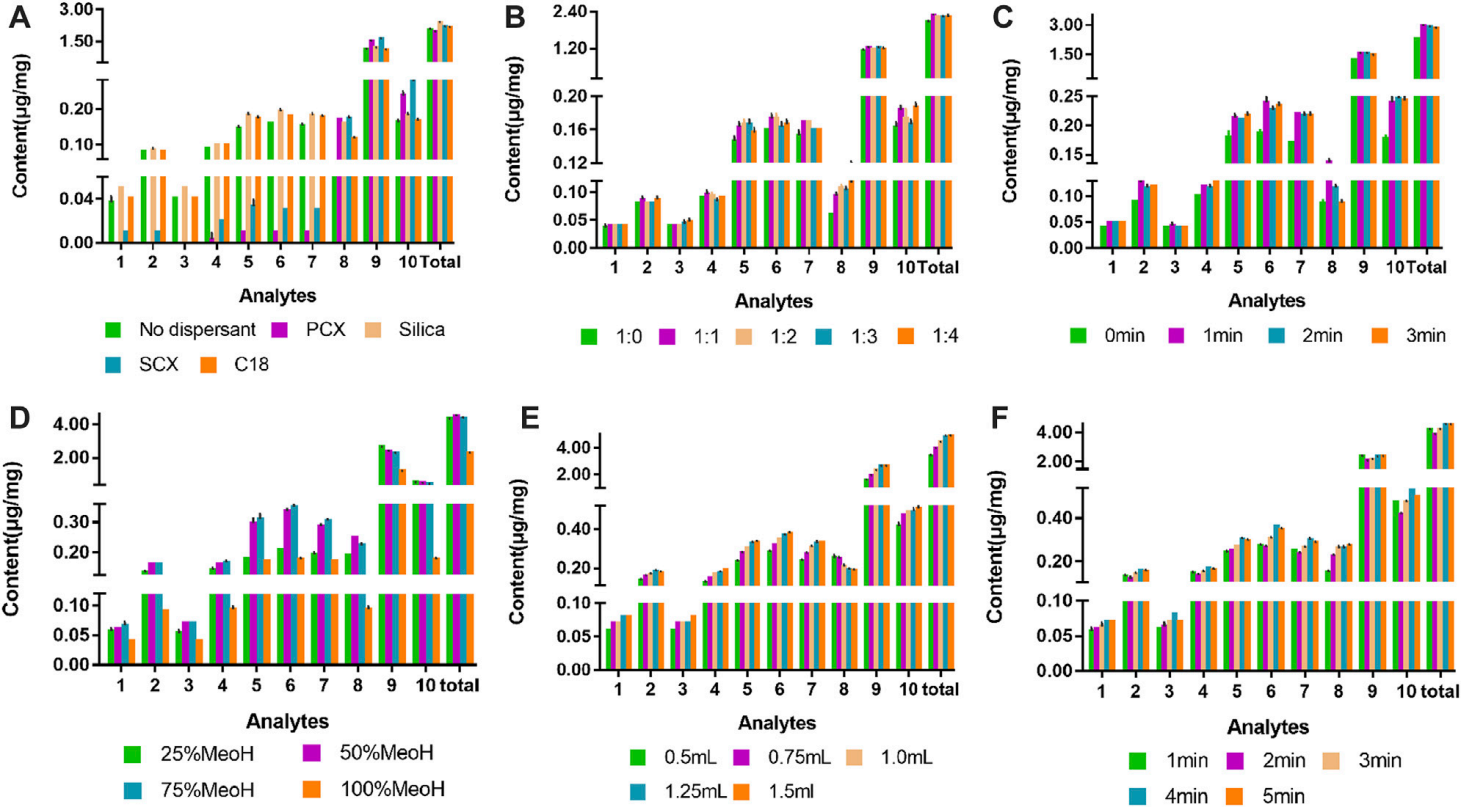
Effect of experimental parameters for the VA-MSPD-UHPLC-MS/MS method on the yield of the ten compounds: **(A)** type of dispersant, **(B)** ratio of sample to dispersant, **(C)** grinding time, **(D)** extraction solvent concentration, **(E)** volume of extraction solvent, and **(F)** vortex time. (1) isocorynoxeine, (2) corynoxeine, (3) isorhychophylline, (4) rhychophylline, (5) geissoschizine methyl ether, (6) hirsuteine, (7) hirsutine, (8) neochlorogenic acid, (9) chlorogenic acid, and (10) cryptochlorogenic acid. “Total” represents the total content of all compounds.

The vital parameter of MSPD is the dispersant, which plays an important role in the blending process. The dispersant not only breaks up the sample structure to expose the target compounds, but also acts as a binding phase to combine with compounds in the sample, facilitating the interaction between the extraction solvent and the sample. In the present study, four types of dispersants (PCX, silica, SCX, and C_18_) were used for optimization of the VA-MSPD. The best total extraction yield of ten active components in Gouteng was achieved when the dispersant used was silica (Figure 3A). The reason for this may be that the hydroxyl groups on the surface of silica formed hydrogen bonds between the extracted components, thereby enhancing the extraction yields. Thus, silica was selected as the dispersant for subsequent analysis.

#### Ratio of sample to dispersant

The amount of dispersant directly affects the strength of the force between the dispersant and the extracted components. In order to ensure a more efficient interaction between the extracted components in the sample and the dispersant, the ratio of the sample to the dispersant (1:0, 1:1, 1:2, and 1:3) was optimized. Each active component exhibited the highest extraction efficiency when the ratio of sample to dispersant was 1:1 (Figure 3B). When the amount of dispersant was increased, the total extraction efficiency of the target compounds did not increase significantly (*p* > 0.05). This phenomenon indicated that when the ratio of sample to dispersant was 1:1, the force between the silica and active compounds reached saturation. Therefore, the ratio of sample to dispersant was set at 1:1.

#### Grinding time

A certain grinding time can increase the contact area between the dispersant and the sample powder, promoting the interaction between the dispersant and the target components. The grinding time (0, 1, 2, and 3 min) was adjusted for optimum extraction efficiency. The highest total extraction yield was obtained with a 1 min grinding time. When the grinding time exceeded 1 min, the total extraction yield of the active components was essentially unchanged (Figure 3C). Hence, the best grinding time was 1 min.

#### Extraction solvent concentration

Methanol was selected as the extraction solvent, and the effects of methanol solutions with different concentrations (v/v) (25%, 50%, 75%, and 100%) on the total extraction efficiency of target components were investigated. The total extraction yield of the seven alkaloids increased with the increase of methanol concentration and reached the maximum when the methanol concentration was 50% (v/v). However, the total extraction yield decreased gradually when the methanol concentration was further increased (Figure 3D). The reason for this is related to the decreased polarity of the extraction solvent. Thus, a 50% (v/v) methanol concentration was considered as the reference value for the BBD-RSM optimization experiment.

#### Volume of extraction solvent

Whether the active components in the sample can be completely extracted depends on the amount of extraction solvent. An appropriate extraction solvent volume can promote the leaching degree of compounds in the sample. The volume of extraction solvent (0.5, 0.75, 1.0, 1.25, and 1.5 mL) was studied in order to improve the extraction yield of the active components. The total yields of the ten active components distinctly increased when the 50% (v/v) methanol solution volume was increased from 0.5 to 1.25 mL. However, there was an insignificant increase when the volume of the 50% (v/v) methanol solution was 1.5 mL (Figure 3E). Therefore, the optimum volume of the extraction solvent was 1.25 mL.

#### Vortex time

Target components in Gouteng were extracted by vortex assisted, and appropriately increasing the vortex time can improve the extraction solvent contact with the sample, thereby increasing the extraction efficiency of the target components. The vortex time (1, 2, 3, 4, and 5 min) was optimized, and the results showed that when the vortex time was set at 4 min, the extraction efficiency of all targets was the best. However, continuously increasing the vortex time did not have a significant positive impact on the total yield of all active components (Figure 3F). Considering the short time consumption, the vortex time was set at 4 min.

#### Response surface optimization experiment

Based on the single factor experiments, the optimal values of each VA-MSPD parameter were acquired. Then, the three key parameters that had a larger effect on the total extraction yield of active components from Gouteng were used for the response surface optimization experiment, including extraction solvent concentration (A), volume of extraction solvent (B), and vortex time (C). Box-Behnken Design was applied for the three-factor-three-level optimization experiment. Seventeen experiments (Supplementary Table S2) were performed using Design Expert (version 8.0.6) software. The experimental results were fitted with a quadratic regression, and the final regression model equation was as follows:

$$Y=+4.80+0.35A+0.093B+0.032C-0.20AB+6.750E-003AC-4.750E-003BC-{0.49A}^{2}-{0.22B}^{2}-{0.24C}^{2}$$

where Y is the expected total content of the ten active ingredients (mg/g). The model goodness of fit was evaluated by analysis of variance (ANOVA) (see Supplementary Table S3). The *p*-value of the model was below 0.0001, demonstrating that the model was successfully established. The “Lack of Fit” (*p* > 0.05) and correlation coefficient (R^2^ = 0.9965) indicated that the regression equation of the model had a good fit with the experimental data. Moreover, the adjusted R^2^ and the predicted R^2^ values were 0.9919 and 0.9758, respectively, and the coefficient of variation (CV) was 0.65%, revealing that the regression of the model was good and the experimental data was accurate and reliable.

**FIGURE 4:**
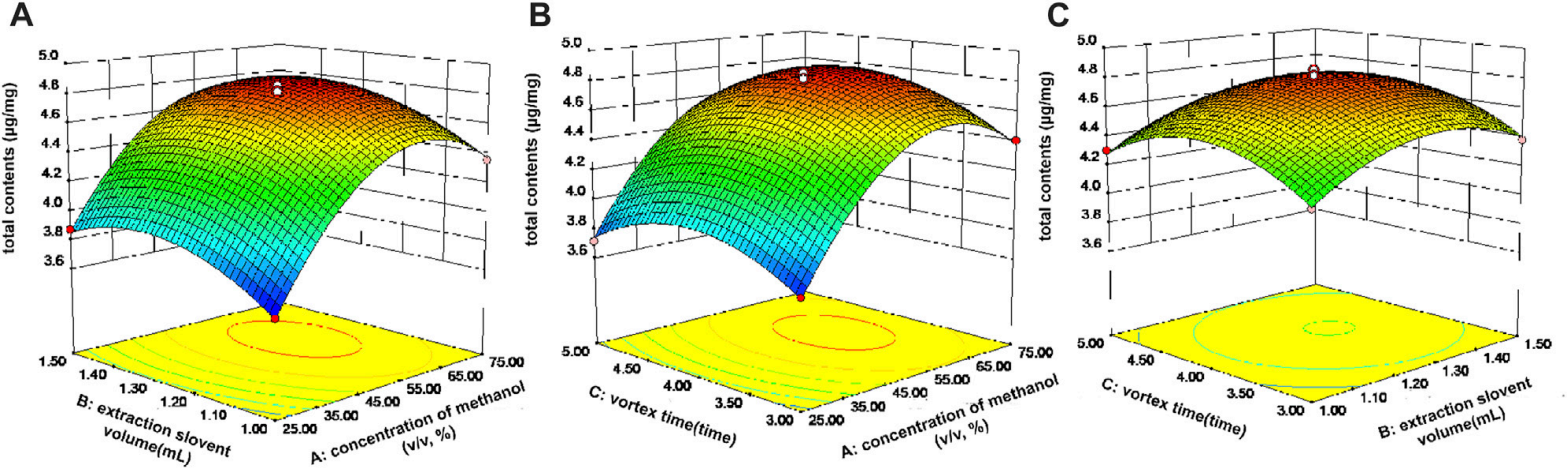
Response surface for the total contents of the ten components in Gouteng. **(A)** Interaction of extraction solvent concentration and extraction solvent volume, **(B)** interaction of extraction solvent concentration and vortex time, and **(C)** interaction of extraction solvent volume and vortex time.

Three dimensional (3D) response surface plots were employed to describe the influences of various factors on the response. Figure 4A depicts the influence of extraction solvent concentration and volume of extraction solvent on the total extraction yields of the ten active components when the vortex time was 4 min. The total extraction yields of all components increased significantly as the extraction solvent concentration increased from 25% to 70% (v/v). At the same time, as the extraction solvent volume increased, the total extraction yields of all components also gradually increased. When the extraction solvent volume exceeded 1.3 mL, the total extraction yields of the active components remained essentially unchanged or even decreased. The total extraction efficiency of all components reached a maximum value when the vortex time was 4 min, which is consistent with the one-factor optimization experiments (Figures 4A,C). Finally, the maximum total content of the ten active ingredients was achieved when the extraction conditions of the VA-MSPD procedure were as follows: extraction solvent at 73.2% (v/v) methanol, the volume of extraction solvent at 1.29 mL, and the vortex time at 4.08 min. The predictive value of the maximum total content of the ten active ingredients was 4.7 mg/g. Subsequently, a validation experiment was carried out, in which the Gouteng power (20 mg) and silica (20 mg) were ground for 1 min and extracted with 1.3 mL of 75% (v/v) methanol for 4 min by vortex. The total extraction content of the ten active ingredients was 4.78 ± 0.03 mg/g, which is close to the predicted value. Therefore, this model was used to convincingly predict the optimal total content of the ten active components in Gouteng.

### Method validation

#### Calibration curve and sensitivity

Ten standard solutions were mixed at the appropriate concentration to obtain a mixed standard solution. The mixed standard solution was diluted stepwise with methanol to obtain standard curve solutions. A calibration curve was established by plotting the peak area ratio of the analyte to IS against concentrations with different weight coefficients (1/X^2^ or 1/X). The correlation coefficients r) of all regression equations were greater than 0.9990, which demonstrates the good linearity within the established ranges (Supplementary Table S4). The concentrations of the target compound at signal to noise ratios (S/N) of 3 and 10 were defined as the limit of detection (LOD) and the limit of quantification (LOQ), respectively. The LOD values of all compounds ranged from 0.10 to 10.7 (ng/mL) and the LOQs between 0.34 and 43.1 (ng/mL).

#### Repeatability, precision and stability

In order to assess the repeatability of the established analytical method, the same sample was processed six times in parallel by the VA-MSPD-UHPLC-MS/MS procedure. The results indicated that relative standard deviations (RSDs) of ten compounds were lower than 2.80%. The mixed standard solutions of three different concentrations were used to evaluate intra-day and inter-day precision, and the results were presented with RSDs, which were all less than 2.97%. The stability was evaluated by measuring the concentration changes of ten compounds at three levels at room temperature for 24 h. The remains of all compounds at three concentration levels were within the range of 87.9%–111% with RSDs below 2.94% (Supplementary Table S5).

#### Recovery

Recovery is an important index and was applied to evaluate the accuracy of the developed VA-MSPD-UHPLC-MS/MS method. A recovery test was performed by assaying the spiked and unspiked samples, where all samples were processed using the optimized VA-MSPD-UHPLC-MS/MS procedure. The range of the average recoveries for all compounds was 95.9%–103% and the corresponding RSDs were all below 2.77% (Supplementary Table S6). The results demonstrate that the developed VA-MSPD-UHPLC-MS/MS method was accurate and reliable.

#### Application

The contents of ten active components in fifteen batches of Gouteng samples were determined by the developed analytical VA-MSPD-UHPLC-MS/MS method. The contents of isocorynoxeine, corynoxeine, isorhychophylline, rhynchophylline, geissoschizine methyl ether, hirsuteine, hirsutine, neochlorogenic acid, chlorogenic acid, and cryptochlorogenic acid in Gouteng were in the range of 0.001–0.951 mg/g, 0.009–1.245 mg/g, 0.024–0.709 mg/g, 0.047–0.737 mg/g, 0.002–0.546 mg/g, 0.004–0.441 mg/g, 0.002–0.439 mg/g, 0.007–0.103 mg/g, 0.070–8.277 mg/g, and 0.020–0.367 mg/g, respectively (Table 1). The contents of the seven alkaloids in different batches of samples were significantly different. According to the literature, the seven alkaloids present properties such as neuroprotection, vasodilator, and antitumor (Shimada et al., 1999; Huang et al., 2021; Meng et al., 2021), and are considered as the main active components of Gouteng. Moreover, the alkaloids are characteristic compounds in Gouteng (Xie et al., 2013). Thus, based on the screening principles of quality markers: 1) the markers are easily attained and accurately quantified; 2) the markers possess significant activity; and 3) the markers are able to precisely distinguish medicinal materials of different quality, the seven alkaloids are suitable for acting as quality markers of Gouteng. For multiple origins of Gouteng, it is difficult to authenticate herbal components of Gouteng by observing the appearance. However, the newly developed VA-MSPD-UHPLC-MS/MS method can be used to extract and determine the content of the ingredients in Gouteng from different origins. It is necessary in the future to clarify differences in chemical composition and to realize the identification of different origins of Gouteng using UHPLC-MS/MS.

**TABLE 1:** The contents of ten compounds in Gouteng from 15 batches (Mean ± SD, n = 3, mg/g).

Batch	Isocorynoxeine	Corynoxeine	Isorhychophylline	Rhynchophylline	Geissoschizine methyl ether	Hirsuteine		Hirsutine	Neochlorogenic acid	Chlorogenic acid	Cryptochlorogenic acid
S1	0.951 ± 0.02	1.245 ± 0.01	0.709 ± 0.01	0.737 ± 0.01	0.033 ± 0.00	0.026 ± 0.00		0.024 ± 0.00	0.075 ± 0.00	8.277 ± 0.15	0.239 ± 0.00
S2	0.427 ± 0.01	0.624 ± 0.01	0.420 ± 0.01	0.484 ± 0.01	0.019 ± 0.00	0.007 ± 0.00		0.008 ± 0.00	0.055 ± 0.00	6.075 ± 0.10	0.133 ± 0.00
S3	0.534 ± 0.01	0.537 ± 0.01	0.350 ± 0.00	0.332 ± 0.01	0.008 ± 0.00	0.070 ± 0.00		0.074 ± 0.00	0.011 ± 0.00	5.148 ± 0.09	0.043 ± 0.00
S4	0.622 ± 0.01	0.765 ± 0.02	0.456 ± 0.01	0.525 ± 0.01	0.008 ± 0.00	0.015 ± 0.00		0.011 ± 0.00	0.039 ± 0.00	5.018 **±** 0.14	0.109 ± 0.00
S5	0.763 ± 0.01	0.840 ± 0.03	0.538 ± 0.00	0.556 ± 0.01	0.010 ± 0.00	0.050 ± 0.00		0.047 ± 0.00	0.051 ± 0.00	8.233 ± 0.18	0.195 ± 0.00
S6	0.076 ± 0.00	0.249 ± 0.00	0.048 ± 0.01	0.218 ± 0.00	0.501 ± 0.00	0.441 ± 0.00		0.439 ± 0.00	0.103 ± 0.00	4.498 ± 0.03	0.367 ± 0.00
S7	0.593 ± 0.02	0.693 ± 0.01	0.491 ± 0.01	0.555 ± 0.01	0.017 ± 0.00	0.035 ± 0.00		0.047 ± 0.00	0.014 ± 0.00	6.985 ± 0.15	0.128 ± 0.00
S8	0.043 ± 0.00	0.051 ± 0.00	0.030 ± 0.00	0.047 ± 0.00	0.546 ± 0.01	0.321 ± 0.00		0.324 ± 0.00	0.007 ± 0.00	1.456 ± 0.02	0.043 ± 0.00
S9	0.739 ± 0.00	1.171 ± 0.01	0.478 ± 0.00	0.631 ± 0.01	0.014 ± 0.00	0.045 ± 0.00		0.033 ± 0.00	0.041 ± 0.00	4.021 ± 0.02	0.099 ± 0.00
S10	0.040 ± 0.00	0.103 ± 0.00	0.025 ± 0.00	0.105 ± 0.00	0.381 ± 0.02	0.366 ± 0.02		0.265 ± 0.01	0.042 ± 0.00	2.843 ± 0.11	0.167 ± 0.00
S11	0.030 ± 0.00	0.072 ± 0.00	0.026 ± 0.00	0.070 ± 0.00	0.307 ± 0.02	0.289 ± 0.01		0.270 ± 0.02	0.046 ± 0.00	2.734 ± 0.20	0.183 ± 0.00
S12	0.001 ± 0.00	0.009 ± 0.00	0.067 ± 0.00	0.077 ± 0.00	0.002 ± 0.00	0.004 ± 0.02		0.002 ± 0.00	0.010 ± 0.00	0.070 ± 0.00	0.020 ± 0.00
S13	0.002 ± 0.00	0.012 ± 0.00	0.081 ± 0.00	0.092 ± 0.00	0.037 ± 0.00	0.036 ± 0.00		0.029 ± 0.00	0.007 ± 0.00	0.309 ± 0.01	0.026 ± 0.00
S14	0.030 ± 0.00	0.088 ± 0.00	0.024 ± 0.00	0.072 ± 0.00	0.422 ± 0.02	0.400 ± 0.01		0.395 ± 0.01	0.030 ± 0.00	1.566 ± 0.02	0.105 ± 0.00
S15	0.030 ± 0.00	0.088 ± 0.00	0.025 ± 0.00	0.079 ± 0.00	0.421 ± 0.00	0.395 ± 0.01		0.367 ± 0.00	0.022 ± 0.00	1.516 ± 0.02	0.096 ± 0.00

#### Comparison of the VA-MSPD-UHPLC-MS/MS method with other methods

The reported analytical method utilizes ultrasonic extraction, reflux extraction, microwave extraction, and so on. In this study, ultrasonic extraction and reflux extraction were applied to extract the active components of Gouteng in different batches, to compare with the proposed VA-MSPD-UHPLC-MS/MS method. The total contents of the ten compounds were lower when reflux extraction was used, and there was no significant difference in the total contents of the ten compounds between the VA-MSPD-UHPLC-MS/MS and ultrasonic extraction methods (Supplementary Figure S2). Furthermore, in order to evaluate the advantages of the VA-MSPD-UHPLC-MS/MS method, it was compared with other reported methods (Table 2). Compared with the reported methods (Xie et al., 2013; Wang et al., 2014; Hou et al., 2018), the proposed VA-MSPD-UHPLC-MS/MS method presents numerous advantages such as a low amount of materials and reagents used, short sample processing and analytical times, and protection of active components from heat damage. Thus, the established method is feasible for the determination of active components in Gouteng. Moreover, the determination method based on VA-MSPD-UHPLC-MS/MS is a bright prospect for determining the components of Chinese patent medicines which contain Gouteng or biological samples. However, it requires further investigation because of the different matrix effects in different backgrounds.

**TABLE 2:** The comparison of the VA-MSPD-UHPLC-MS/MS with other methods.

No.	Extracted compounds	Sample amounts (g)	Type of solvent	Solvent volume (mL)	Extraction method	Extraction time (min)	Analytical method	Analytical time (min)	References
1	chlorogenic acid, isocorynoxeine, rhynchophylline, isorhynchophylline, corynoxeine	0.2	70% methanol	8	ultrasonic extraction	45	HPLC-DAD	50	Xie et al. (2013)
2	isocorynoxeine, corynoxeine, isorhynchophylline, rhynchophylline, hirsuteine, hirsutine, geissoschizine methyl ether	0.5	MeOH: CH_2_Cl_2_ (1 : 1, v/v)	50	ultrasonic extraction	30	HPLC-DAD	55	Hou et al. (2018)
3	alkaloids	0.2	75% methanol	10	ultrasonic extraction	30	UPLC-ESI-Q-TOF/MS	10.5	Wang et al. (2014)
4	isocorynoxeine, corynoxeine, isorhynchophylline, rhynchophylline	100	70% ethanol	500	reflux	60	HPLC-DAD	50	Lu et al. (2012)
5	isocorynoxeine, corynoxeine, isorhychophylline, rhynchophylline, geissoschizine methyl ether, hirsuteine, hirsutine, chlorogenic acid, neochlorogenic acid, cryptochlorogenic acid	0.02	75% methanol	1.3	VA-MSPD	4	UHPLC-MS/MS	13	This work

## Conclusion

An efficient and simple VA-MSPD-UHPLC-MS/MS method was established and successfully applied to determinate seven alkaloids and three organic acids in Gouteng. Silica was selected as the dispersant in the VA-MSPD-UHPLC-MS/MS procedure. The best extraction yield for all active components in Gouteng was obtained when the ratio of sample to dispersant was 1:1, the grinding time was 1 min, 75% (v/v) methanol was used as the extraction solvent, the volume of extraction solvent was 1.3 mL, and the vortex time was 4 min. Moreover, total sample extraction and analysis only took 18 min. Compared with reported methods, the current analytical method exhibits many merits, such as lower consumption of solvents and materials and a shorter sample processing time. Briefly, the developed VA-MSPD-UHPLC-MS/MS method is a very sensitive, green, and environment-friendly method for the determination of the active ingredients in Gouteng.

## Data Availability

The original contributions presented in the study are included in the article/Supplementary Material, further inquiries can be directed to the corresponding authors.
