# Supplementary material for: Simultaneous extraction and determination of alkaloids and organic acids in Uncariae Ramulas Cum Unicis by vortex-assisted matrix solid phase dispersion extraction coupled with UHPLC-MS/MS
Source: Front Chem. 2023 Jan 25;11:1100150. doi: 10.3389/fchem.2023.1100150 (PMC9912122; doi:10.3389/fchem.2023.1100150)

**Simultaneous extraction and determination of alkaloids and organic acids in *Uncariae Ramulas Cum Unicis* by vortex-assisted matrix solid phase dispersion extraction coupled with UHPLC-MS/MS**

**Xianjun Xu^1^, Jiake Wen^1,2#^, Shuangqi Wang^2^, Jia Hao^2*^, Kunze Du^2^, Shiming Fang^2^, Jun He^2^, Jin Li^2^, Yanxu Chang^2,3*^**

^1^ Wuyishan Institute of biology fujian province, Nanping, Fujian 354300

^2^ State Key Laboratory of Component-based Chinese Medicine, Tianjin University of Traditional Chinese Medicine, Tianjin, 301617, China

^3^Haihe Laboratory of Modern Chinese Medicine, Tianjin 301617, China

^#^ The author contributes equally to first author in this work.

*** Correspondence:**

Yanxu Chang, Tianjin State Key Laboratory of Modern Chinese Medicine, Tianjin University of Traditional Chinese Medicine

E-mail: haojiatjtcm@126.com and Tcmcyx@126.com (Y.x. Chang)





**Figure S1** The structures of the ten chemical components in Gouteng

**Table S1. MRM parameters of positive and negative ion mode analyte and internal standard**

| compounds | t_R_(min) | MRM parameters | | | | | |
| --- | --- | --- | --- | --- | --- | --- | --- |
|  |  | Q1 | Q3 | DP(V) | EP(V) | CE(V) | CXP(V) |
| Isocorynoxeine | 6.63 | 383.1 | 160.2 | 65 | 6 | 43 | 6 |
| Corynoxeine | 7.06 | 383.0 | 267.2 | 63 | 6 | 40 | 7 |
| Isorhychophylline | 6.97 | 385.1 | 241.1 | 60 | 10 | 41 | 9 |
| rhychophylline | 7.56 | 385.3 | 160.2 | 55 | 7 | 44 | 6 |
| Geissoschizine methyl ether | 10.39 | 367.3 | 144.0 | 55 | 6 | 38 | 6 |
| Hirsuteine | 10.82 | 367.1 | 170.2 | 62 | 4 | 35 | 10 |
| Hirsutine | 11.57 | 369.3 | 144.2 | 64 | 6 | 42 | 5 |
| Neochlorogenic acid | 1.76 | 353.0 | 191.0 | -38 | -4 | -25 | -4 |
| Chlorogenic acid | 2.81 | 353.1 | 190.8 | -30 | -6 | -22 | -10 |
| Cryptochlorogenic acid | 3.06 | 353.0 | 173.1 | -40 | -4 | -22 | -4 |
| Nuciferine (IS) | 7.64 | 296.1 | 265.1 | 38 | 5 | 20 | 8 |
| Rosmarinic acid (IS) | 7.05 | 359.0 | 161.0 | -33 | -6 | -23 | -9 |

**Table S2. Box-Benhnken design and experimental/predicted values of total contents of analytes**

| Run | A (%, v/v) | B (mL) | C (min) | Total contents of analytes (µg/mg) | Predicted  (µg/mg) |
| --- | --- | --- | --- | --- | --- |
| 1 | 50 | 1.50 | 3 | 4.379 | 4.401 |
| 2 | 75 | 1.00 | 4 | 4.353 | 4.369 |
| 3 | 50 | 1.00 | 5 | 4.302 | 4.279 |
| 4 | 50 | 1.25 | 4 | 4.852 | 4.797 |
| 5 | 50 | 1.25 | 4 | 4.814 | 4.797 |
| 6 | 50 | 1.25 | 4 | 4.796 | 4.797 |
| 7 | 50 | 1.00 | 3 | 4.194 | 4.206 |
| 8 | 25 | 1.25 | 3 | 3.697 | 3.690 |
| 9 | 25 | 1.25 | 5 | 3.713 | 3.740 |
| 10 | 25 | 1.00 | 4 | 3.634 | 3.629 |
| 11 | 50 | 1.25 | 4 | 4.767 | 4.797 |
| 12 | 50 | 1.25 | 4 | 4.755 | 4.797 |
| 13 | 25 | 1.50 | 4 | 3.870 | 3.854 |
| 14 | 75 | 1.50 | 4 | 4.510 | 4.515 |
| 15 | 75 | 1.25 | 3 | 4.404 | 4.377 |
| 16 | 50 | 1.50 | 5 | 4.468 | 4.456 |
| 17 | 75 | 1.25 | 5 | 4.447 | 4.454 |

A: concentration of methanol; B: extraction solvent volume; C: vortex time

**Table S3. ANOVA analysis of BBD-RSM regression model**

| Source | Sum of Squares | df | Mean Square | F-Value | *p*-value | significant |
| --- | --- | --- | --- | --- | --- | --- |
| Model | 2.65 | 9 | 0.29 | 218.74 | < 0.0001 | ** |
| A | 0.98 | 1 | 0.98 | 728.21 | < 0.0001 | ** |
| B | 0.069 | 1 | 0.069 | 51.41 | 0.0002 | ** |
| C | 8.19E-03 | 1 | 8.19E-03 | 6.09 | 0.043 | * |
| AB | 1.56E-03 | 1 | 1.56E-03 | 1.16 | 0.3173 |  |
| AC | 1.82E-04 | 1 | 1.82E-04 | 0.14 | 0.7237 |  |
| BC | 9.03E-05 | 1 | 9.03E-05 | 0.067 | 0.8031 |  |
| A2 | 1 | 1 | 1 | 744.4 | < 0.0001 | ** |
| B2 | 0.2 | 1 | 0.2 | 147.7 | < 0.0001 | ** |
| C2 | 0.25 | 1 | 0.25 | 185.93 | < 0.0001 | ** |
| Residual | 9.42E-03 | 7 | 1.35E-03 |  |  |  |
| Lack of Fit | 3.44E-03 | 3 | 1.15E-03 | 0.77 | 0.569 | not significant |
| Pure Error | 5.98E-03 | 4 | 1.50E-03 |  |  |  |
| Cor Total | 2.66 | 16 |  |  |  |  |
| R-Squared | 0.9965 |  |  |  |  |  |
| 1. Squared   Adjusted | 0.9919 |  |  |  |  |  |
| 1. Squared   Predict | 0.9758 |  |  |  |  |  |
| CV % | 0.84 |  |  |  |  |  |

Factor A: concentration of methanol solution; B: extraction solvent volume; C: vortex time; ** indicate the level of significance at *P* < 0.01, * indicate the level of significance at *P* < 0.05.

**Table S4. The regressive equations, linear ranges, LOQ and LOD of ten target analytes**

| Analytes | Regressive equation | r | Linear range (ng/mL) | Weighting | Accuracy (%) | RSD  (%) | LOQ (ng/mL) | LOD  (ng/mL) |
| --- | --- | --- | --- | --- | --- | --- | --- | --- |
| Isocorynoxeine | y=0.00189x+0.0085 | 0.9992 | 5.00-1000 | 1/(x*x) | 100 | 3.54 | 0.87 | 0.26 |
| Corynoxeine | y=0.00024x+0.00435 | 0.9993 | 20.0-4000 | 1/(x*x) | 99.9 | 6.63 | 0.60 | 0.18 |
| Isorhychophylline | y=0.000484x+0.00106 | 0.9990 | 2.50-1000 | 1/(x*x) | 100 | 4.08 | 0.50 | 0.15 |
| Rhynchophylline | y=0.00126x+0.023 | 0.9994 | 20.0-4000 | 1/(x*x) | 99.9 | 3.00 | 0.34 | 0.10 |
| Geissoschizine methyl ether | y=0.000874x+0.00674 | 0.9990 | 20.0-4000 | 1/(x*x) | 100 | 3.65 | 0.58 | 0.17 |
| Hirsuteine | y=0.000253x+0.000845 | 0.9998 | 10.0-4000 | 1/x | 100 | 3.75 | 1.17 | 0.35 |
| Hirsutine | y=0.00121x+0.0217 | 0.9990 | 40.0-4000 | 1/x | 99.9 | 5.42 | 0.39 | 0.12 |
| Neochlorogenic acid | y=0.000108x+0.00125 | 0.9994 | 100-1000 | 1/(x*x) | 99.9 | 2.00 | 43.1 | 10.7 |
| Chlorogenic acid | y=0.00154x+0.0531 | 0.9992 | 100-20000 | 1/(x*x) | 99.9 | 3.46 | 15.4 | 4.61 |
| Cryptochlorogenic acid | y=0.000935x+0.00241 | 0.9997 | 20.0-4000 | 1/(x*x) | 100 | 2.30 | 6.89 | 2.07 |

**Table S5. The precision, accuracy, repeatability and stability of ten target analytes (n = 6)**

| Analytes | Concentration (ng/mL) | Intra-day | | Inter-day | | Stability | | Repeatability |
| --- | --- | --- | --- | --- | --- | --- | --- | --- |
|  |  | RSD (%) | Accuracy (%) | RSD (%) | Accuracy (%) | RSD (%) | Remain (%) | RSD (%) |
| Isocorynoxeine | 50 | 0.95 | 111 | 1.79 | 112 | 2.94 | 108 | 1.73 |
|  | 500 | 1.21 | 106 | 2.79 | 104 | 2.12 | 104 |  |
|  | 800 | 2.01 | 88.4 | 1.95 | 88.3 | 2.88 | 88.3 |  |
| Corynoxeine | 200 | 1.72 | 111 | 2.94 | 111 | 1.55 | 113 | 1.04 |
|  | 2000 | 1.30 | 101 | 2.17 | 102 | 1.92 | 104 |  |
|  | 3200 | 1.13 | 102 | 2.58 | 98.9 | 2.31 | 94.4 |  |
| Isorhychophylline | 50 | 2.28 | 93.8 | 2.70 | 93.3 | 2.64 | 94.4 | 2.80 |
|  | 500 | 2.07 | 96.1 | 2.80 | 95.7 | 2.89 | 101 |  |
|  | 800 | 1.38 | 86.1 | 2.23 | 87.0 | 2.38 | 91.4 |  |
| Rhynchophylline | 200 | 0.72 | 113 | 1.80 | 112 | 1.60 | 111 | 1.31 |
|  | 2000 | 1.75 | 91.7 | 2.50 | 93.8 | 2.71 | 93.5 |  |
|  | 3200 | 1.18 | 85.3 | 1.34 | 85.2 | 1.45 | 87.9 |  |
| Geissoschizine methyl ether | 200 | 2.60 | 108 | 2.37 | 108 | 2.85 | 109 | 1.93 |
|  | 2000 | 1.24 | 112 | 2.62 | 111 | 2.27 | 108 |  |
|  | 3200 | 1.60 | 96.9 | 2.53 | 94.8 | 2.90 | 92.1 |  |
| Hirsuteine | 200 | 2.58 | 109 | 2.43 | 110 | 2.41 | 109 | 2.36 |
|  | 2000 | 1.78 | 106 | 2.56 | 108 | 2.04 | 111 |  |
|  | 3200 | 2.97 | 98.8 | 2.70 | 97.4 | 0.90 | 93.8 |  |
| Hirsutine | 200 | 1.28 | 106 | 2.80 | 110 | 2.48 | 111 | 2.75 |
|  | 2000 | 1.19 | 106 | 1.87 | 106 | 2.62 | 100 |  |
|  | 3200 | 1.36 | 97.0 | 2.61 | 94.0 | 1.73 | 91.1 |  |
| Neochlorogenic acid | 50 | 2.08 | 96.6 | 2.39 | 96.4 | 2.26 | 104 | 1.96 |
|  | 500 | 2.25 | 90.1 | 2.41 | 90.5 | 2.48 | 91.7 |  |
|  | 800 | 1.32 | 94.3 | 1.53 | 93.2 | 1.79 | 93.4 |  |
| Chlorogenic acid | 1000 | 1.56 | 105 | 2.21 | 104 | 2.65 | 105 | 1.81 |
|  | 10000 | 1.13 | 101 | 2.05 | 99.3 | 2.09 | 96.3 |  |
|  | 16000 | 1.37 | 97.4 | 2.45 | 95.4 | 2.14 | 95.2 |  |
| Cryptochlorogenic acid | 200 | 1.74 | 98.4 | 2.33 | 97.0 | 1.68 | 103 | 2.19 |
|  | 2000 | 1.58 | 113 | 2.38 | 110 | 2.38 | 109 |  |
|  | 3200 | 1.85 | 104 | 2.15 | 103 | 1.67 | 99.0 |  |

| Analytes | | Sample  (ng mL-1) | | Spiked  (ng mL-1) | | Recovery  (%) | | RSD  (%) |
| --- | --- | --- | --- | --- | --- | --- | --- | --- |
| Isocorynoxeine | 150.4 | | 151.0 | | 99.4 | | 1.64 | |
| Corynoxeine | 489.1 | | 490.0 | | 103 | | 2.26 | |
| Isorhychophylline | 94.3 | | 94.5 | | 98.0 | | 2.05 | |
| Rhynchophylline | 465.7 | | 466.0 | | 95.9 | | 2.09 | |
| Geissoschizine methyl ether | 1068.3 | | 1069.0 | | 96.3 | | 0.70 | |
| Hirsuteine | 959.2 | | 960.0 | | 96.5 | | 1.00 | |
| Hirsutine | 912.5 | | 913.0 | | 99.0 | | 1.66 | |
| Neochlorogenic acid | 315.3 | | 316.0 | | 98.8 | | 2.75 | |
| Chlorogenic acid | 9658.3 | | 9660.0 | | 96.1 | | 2.77 | |
| Cryptochlorogenic acid | 888.0 | | 880.0 | | 102 | | 1.88 | |

**Table S6. The recoveries of ten target analytes**

**Figure S2** The comparison of the VA-MSPD-UHPLC-MS/MS method with other methods in the determination of compounds in three different batches of Gouteng


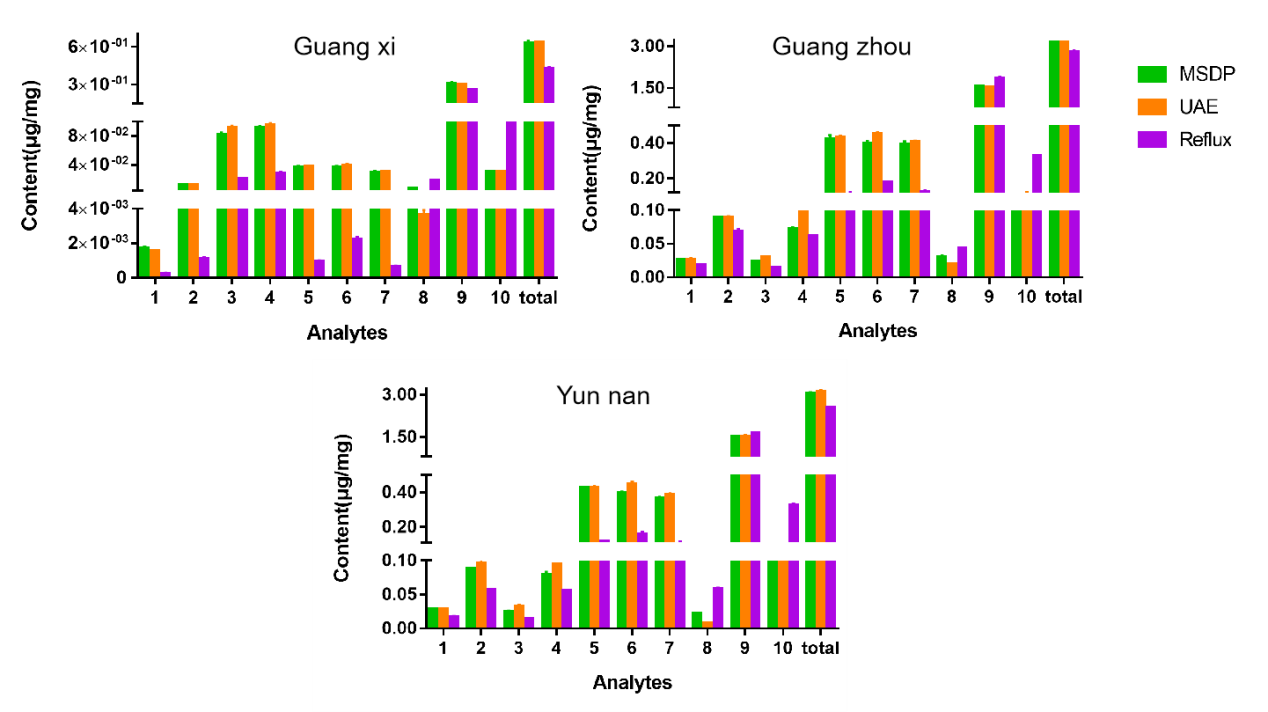

Supplement: Supplementary file 1 [file DataSheet1.docx]
